# Supplementary material for: MetaRibo-Seq measures translation in microbiomes
Source: Nat Commun. 2020 Jun 29;11:3268. doi: 10.1038/s41467-020-17081-z (PMC7324362; doi:10.1038/s41467-020-17081-z)
Supplement: Supplementary file 10 — Supplementary Data 7 [file 41467_2020_17081_MOESM10_ESM.zip › File2/Confidence_High_Taxonomy/395494_out.krona.html]

Javascript must be enabled to view this page.

members
magnitude
magnitudeUnassigned
count
unassigned
taxon
rank

395494\_out

136

superkingdom
2
136

1239
136
phylum

class
186801
136


SRS022713\_contig\_number\_2005SRS075078\_contig\_number\_27401SRS104197\_contig\_number\_contig-100\_1256.1256SRS147614\_contig\_number\_contig-100\_707.68918
order
4
136
186802


SRS056259\_contig\_number\_contig-100\_50.175635
186803
83
1
family

1
28050
genus

species

SRS050925\_contig\_number\_contig-100\_655.198352
28052
1

species

SRS013476\_contig\_number\_606
1898203
1


SRS012969\_contig\_number\_41199SRS013687\_contig\_number\_contig-100\_449.197731SRS013951\_contig\_number\_contig-100\_995.225917SRS014736\_contig\_number\_9331SRS014923\_contig\_number\_contig-100\_657.247134SRS015217\_contig\_number\_15239SRS015890\_contig\_number\_contig-100\_570.163740SRS016095\_contig\_number\_29653SRS017307\_contig\_number\_contig-100\_1348.182888SRS018623\_contig\_number\_contig-100\_769.132898SRS018656\_contig\_number\_12335SRS018817\_contig\_number\_contig-100\_625.147146SRS018836\_contig\_number\_contig-100\_482.167165SRS019267\_contig\_number\_contig-100\_616.63839SRS019601\_contig\_number\_24979SRS023715\_contig\_number\_contig-100\_554.176329SRS024075\_contig\_number\_contig-100\_249.109135SRS043411\_contig\_number\_contig-100\_813.109725SRS043768\_contig\_number\_contig-100\_492.192625SRS045826\_contig\_number\_contig-100\_1653.124111SRS047044\_contig\_number\_17872SRS047433\_contig\_number\_contig-100\_346.141382SRS050941\_contig\_number\_18767SRS050998\_contig\_number\_17390SRS051882\_contig\_number\_22740SRS052697\_contig\_number\_46738SRS053398\_contig\_number\_contig-100\_574.163708SRS055982\_contig\_number\_contig-100\_1580.109406SRS056273\_contig\_number\_contig-100\_1134.204494SRS057478\_contig\_number\_9053SRS063040\_contig\_number\_8101SRS063190\_contig\_number\_15359SRS075021\_contig\_number\_contig-100\_536.61133SRS075984\_contig\_number\_14369SRS076756\_contig\_number\_contig-100\_288.140711SRS077335\_contig\_number\_1660SRS1041033\_contig\_number\_12826SRS1041136\_contig\_number\_27067SRS104485\_contig\_number\_contig-100\_981.44786SRS1055067\_contig\_number\_16153SRS140492\_contig\_number\_34643SRS142542\_contig\_number\_26698SRS143372\_contig\_number\_contig-100\_829.89333SRS143523\_contig\_number\_contig-100\_311.159955SRS144537\_contig\_number\_48778SRS147377\_contig\_number\_contig-100\_889.80910SRS147766\_contig\_number\_2815SRS148874\_contig\_number\_16478SRS149784\_contig\_number\_contig-100\_602.172164
species
49
39491


SRS055017\_contig\_number\_contig-100\_1269.209667
genus
1
841
31


SRS014459\_contig\_number\_24320SRS077194\_contig\_number\_contig-100\_646.192666SRS1055049\_contig\_number\_10867
species
2293137
3

166486
3

SRS019445\_contig\_number\_37635SRS144506\_contig\_number\_54311SRS149075\_contig\_number\_19936
species


SRS140513\_contig\_number\_16015
species
1
2293144

4
301302

SRS014855\_contig\_number\_18022SRS016267\_contig\_number\_contig-100\_725.87734SRS077552\_contig\_number\_contig-100\_269.175671SRS143417\_contig\_number\_29397
species

17
360807
species

SRS016018\_contig\_number\_17446SRS016954\_contig\_number\_contig-100\_332.160028SRS016989\_contig\_number\_contig-100\_429.101773SRS018575\_contig\_number\_4958SRS019693\_contig\_number\_contig-100\_393.72314SRS021948\_contig\_number\_contig-100\_531.206850SRS046369\_contig\_number\_contig-100\_1084.108065SRS047014\_contig\_number\_24272SRS064645\_contig\_number\_contig-100\_197.25504SRS1041118\_contig\_number\_5217SRS104311\_contig\_number\_contig-100\_2107.252210SRS1054716\_contig\_number\_contig-100\_1041.91295SRS142712\_contig\_number\_contig-100\_330.50521SRS147557\_contig\_number\_contig-100\_1172.196722SRS148253\_contig\_number\_14610SRS893270\_contig\_number\_contig-100\_318.134751SRS893327\_contig\_number\_contig-100\_301.76599

species

SRS1041032\_contig\_number\_12001
2049040
1

1
2293127
species

SRS143466\_contig\_number\_30173

1
384638

SRS019381\_contig\_number\_22103
species

family
48
31979

genus
48
1485

species

SRS013965\_contig\_number\_contig-100\_1070.223987SRS014613\_contig\_number\_contig-100\_772.124472SRS014948\_contig\_number\_23544SRS014979\_contig\_number\_contig-100\_1547.180859SRS015854\_contig\_number\_24889SRS016335\_contig\_number\_44208SRS017521\_contig\_number\_contig-100\_695.267513SRS019161\_contig\_number\_contig-100\_652.242935SRS019787\_contig\_number\_14050SRS020869\_contig\_number\_39223SRS022609\_contig\_number\_contig-100\_477.273957SRS023526\_contig\_number\_contig-100\_1262.222109SRS023583\_contig\_number\_15145SRS043701\_contig\_number\_17913SRS043841\_contig\_number\_contig-100\_360.83085SRS044535\_contig\_number\_20460SRS049446\_contig\_number\_15657SRS050974\_contig\_number\_contig-100\_1172.1173SRS054905\_contig\_number\_contig-100\_915.147367SRS058723\_contig\_number\_3340SRS063127\_contig\_number\_contig-100\_577.40107SRS065504\_contig\_number\_38339SRS075773\_contig\_number\_39204SRS075821\_contig\_number\_contig-100\_1633.140873SRS077231\_contig\_number\_26639SRS077502\_contig\_number\_contig-100\_1903.95217SRS077641\_contig\_number\_contig-100\_465.136876SRS078242\_contig\_number\_contig-100\_1155.193285SRS097889\_contig\_number\_contig-100\_373.228027SRS1041134\_contig\_number\_contig-100\_1533.86378SRS1041145\_contig\_number\_contig-100\_433.196451SRS104400\_contig\_number\_contig-100\_436.346034SRS104693\_contig\_number\_contig-100\_304.96926SRS104975\_contig\_number\_25325SRS1055056\_contig\_number\_14291SRS142503\_contig\_number\_53358SRS142980\_contig\_number\_contig-100\_366.56143SRS143342\_contig\_number\_contig-100\_1064.139276SRS143991\_contig\_number\_contig-100\_1064.170116SRS144362\_contig\_number\_35143SRS144714\_contig\_number\_contig-100\_938.173320SRS145308\_contig\_number\_contig-100\_956.38330SRS147139\_contig\_number\_contig-100\_988.243117SRS147425\_contig\_number\_contig-100\_160.85582SRS147977\_contig\_number\_contig-100\_402.92170SRS148721\_contig\_number\_contig-100\_977.313613SRS148817\_contig\_number\_contig-100\_729.94627
2293030
47

1
59620

SRS049896\_contig\_number\_34470
species
